# Supplementary material for: Variation in Water-Holding Capacity in Sphagnum Species Depends on Both Plant and Colony Structure
Source: Plants (Basel). 2024 Apr 9;13(8):1061. doi: 10.3390/plants13081061 (PMC11053561; doi:10.3390/plants13081061)
Supplement: Supplementary file 1 [file plants-13-01061-s001.zip › plants-2895679-1-supplementary.pdf]

Supplementary material

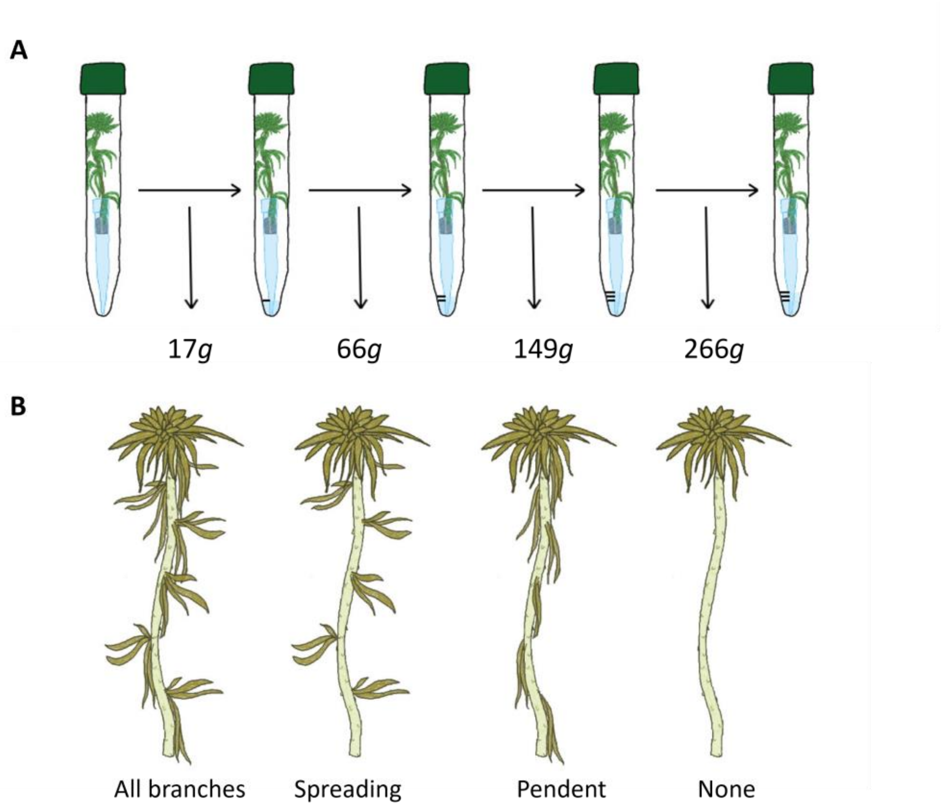

Figure S1. A. Diagram illustrating the set up for water removal by centrifugation (please see methods for details). **B.** Diagram illustrating branch removal of *Sphagnum* plants before centrifugation.

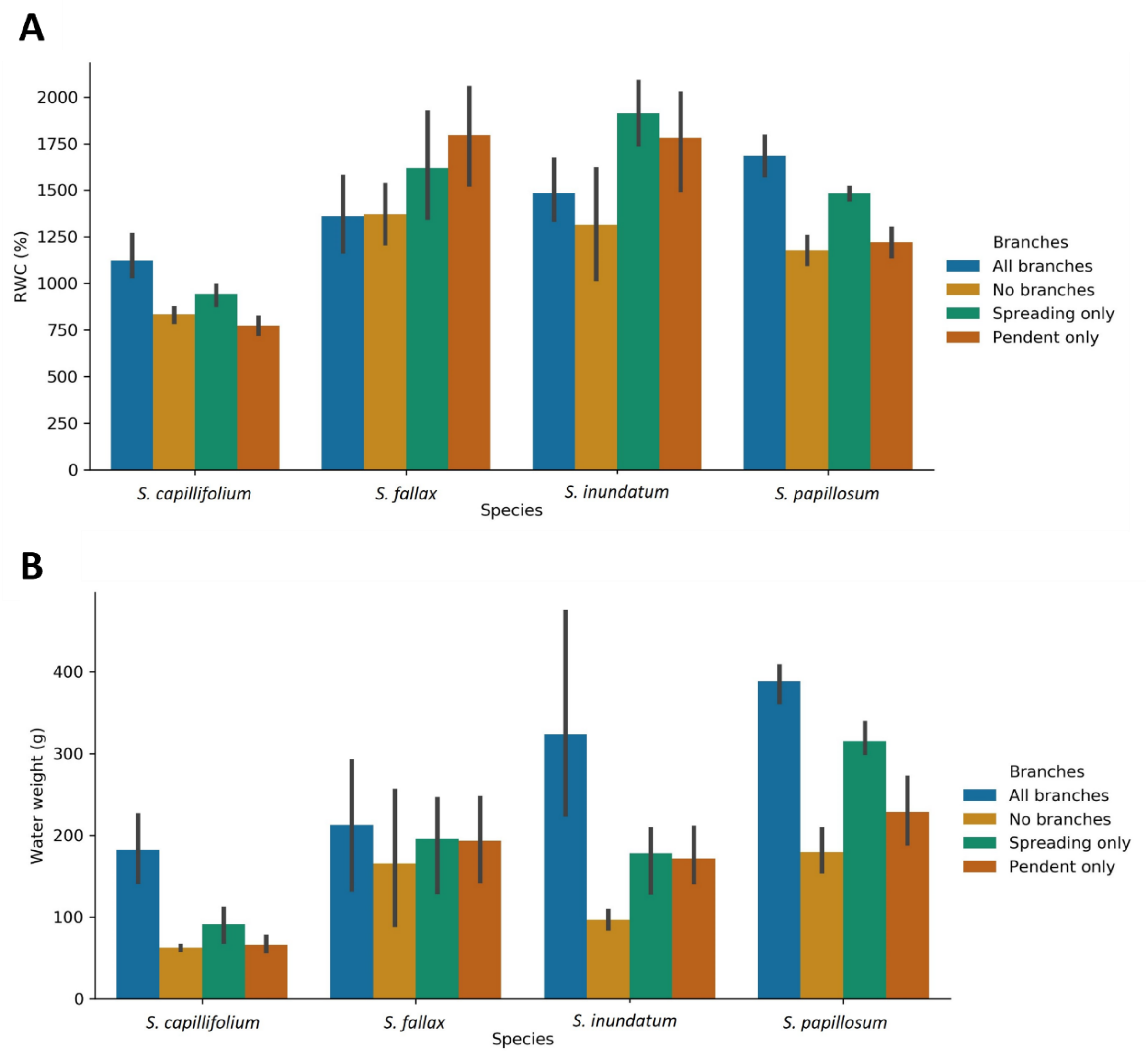

Figure S2. A. Plant water content, expressed in grams of water per gram of dry weight, is not affected by branch removal for the four *Sphagnum* species as indicated. **B.** Total amount of water stored in whole plants or plants where the branches have been selectively removed as indicated.

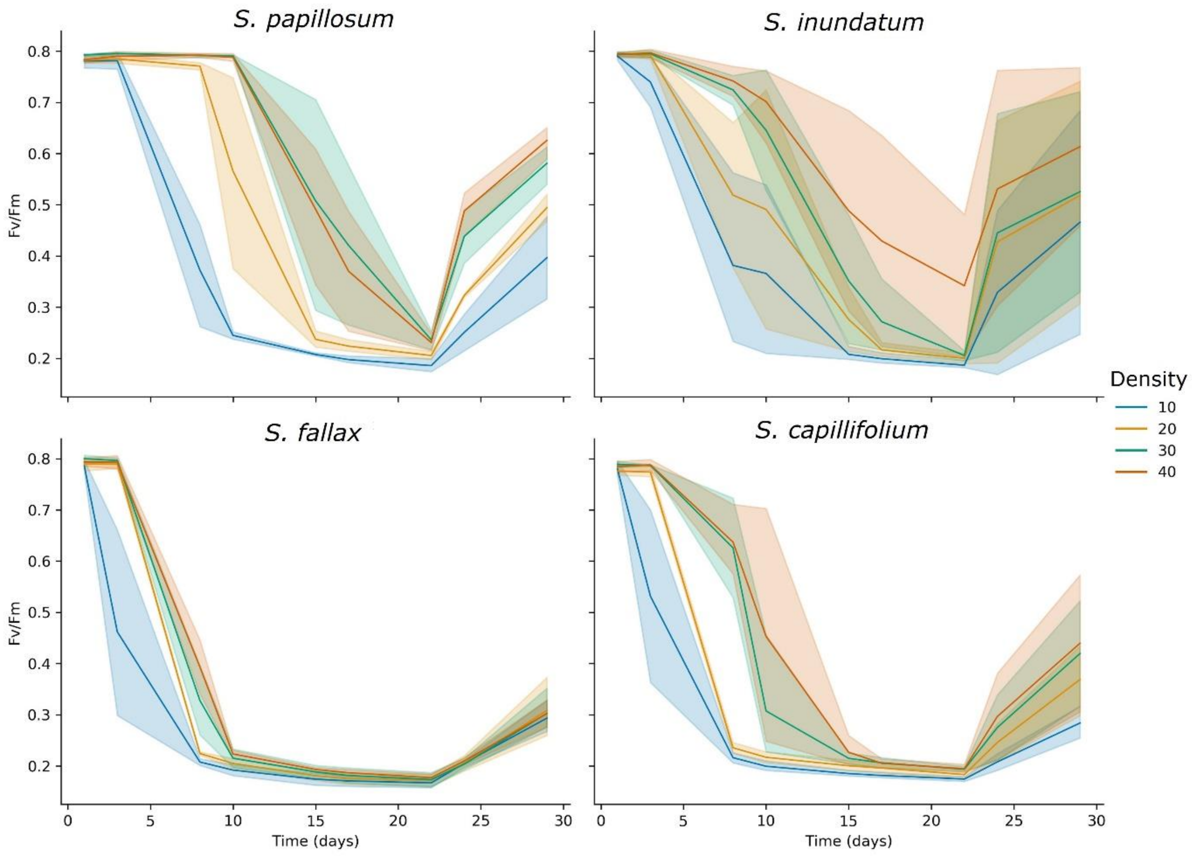

Figure S3. Effect of manipulated canopy density on plant heath as measured by the chlorophyll fluoresce index *Fv/Fm* during an imposed drought scenario. Plants in cosms at the indicated stem density for each species were subjected to water withdrawal for 21 days after which they were re-watered. *Fv/Fm* was calculated at different time points during the experiment. Lines represent the mean and the shaded areas the 95% confidence interval for each sample.

Table S1: Statistical analysis for the data presented in Figures 2 and 3.

Figure 2A. Within species comparison

	g	0	17	66	149	266
S. capillifoliun	300	ns	-	ns	ns	*
	600	**	ns	-	ns	ns
	900	**	ns	ns	-	ns
	1500	**	*	ns	ns	-
S. fallax	300	ns	-	ns	ns	ns
	600	*	ns	-	ns	ns
	900	*	ns	ns	-	ns
	1500	**	ns	ns	ns	-
S. inundatum	300	**	-	**	**	**
	600	**	**	-	ns	ns
	900	**	**	ns	-	ns
	1500	**	**	ns	ns	-
S. papillosum	300	**	-	**	**	**
	600	**	**	-	ns	ns
	900	**	**	ns	-	ns
	1500	**	**	ns	ns	-

Figure 2A. Between species comparison

		S. capillifc	S. fallax	S. inundat	S. papillosum
17*g*	S. capillifoliun	-	ns	*	**
	S. fallax	ns	-	ns	*
	S. inundatum	*	ns	-	*
	S. papillosum	**	*	*	-
66*g*	S. capillifoliun	-	ns	**	**
	S. fallax	ns	-	ns	**
	S. inundatum	**	ns	-	*
	S. papillosum	**	**	*	-
149*g*	S. capillifoliun	-	ns	**	**
	S. fallax	ns	-	ns	**
	S. inundatum	**	ns	-	**
	S. papillosum	**	**	**	-
266*g*	S. capillifoliun	-	ns	**	**
	S. fallax	ns	-	ns	**
	S. inundatum	**	ns	-	**
	S. papillosum	**	**	**	-

Figure 3A. No Branches

	g	0	17	66	149	266
S. capillifoliun	300	ns	-	ns	*	**
	600	ns	ns	-	ns	ns
	900	*	*	ns	-	ns
	1500	**	**	ns	ns	-
S. fallax	300	ns	-	*	*	*
	600	**	*	-	ns	ns
	900	**	*	ns	-	ns
	1500	**	*	ns	ns	-
S. inundatum	300	*	-	**	**	**
	600	**	**	-	ns	*
	900	**	**	ns	-	
	1500	**	**	*	ns	
S. papillosum	300	*	-	*	*	*
	600	**	*	-	ns	ns
	900	**	*	ns	-	ns
	1500	**	*	ns	ns	-

Figure 3B. Spreading only

	g	0	17	66	149	266
S. capillifoliun	300	ns	-	*	*	**
	600	*	*	-	ns	ns
	900	*	*	ns	-	ns
	1500	**	**	ns	ns	-
S. fallax	300	**	-	ns	ns	ns
	600	**	ns	-	ns	ns
	900	**	ns	ns	-	ns
	1500	**	ns	ns	ns	-
S. inundatum	300	**	-	**	**	**
	600	**	**	-	ns	ns
	900	**	**	ns	-	ns
	1500	**	**	ns	ns	-
S. papillosum	300	**	-	**	**	**
	600	**	**	-	ns	ns
	900	**	**	ns	-	ns
	1500	**	**	ns	ns	-

Figure 3C. Pendent only

	g	0	17	66	149	266
S. capillifoliun	300	ns	-	ns	**	**
	600	ns	ns	-	*	*
	900	**	**	*	-	ns
	1500	**	**	*	ns	-
S. fallax	300	**	-	ns	ns	ns
	600	**	ns	-	ns	ns
	900	**	ns	ns	-	ns
	1500	**	ns	ns	ns	-
S. inundatum	300	**	-	ns	ns	ns
	600	**	ns	-	ns	ns
	900	**	ns	ns	-	ns
	1500	**	ns	ns	ns	-
S. papillosum	300	**	-	ns	**	**
	600	**	ns	-	ns	ns
	900	**	**	ns	-	ns
	1500	**	**	ns	ns	-
